# Amoxicillin impact on pathophysiology induced by short term high salt diet in mice

**DOI:** 10.1038/s41598-022-21270-9

**Published:** 2022-11-11

**Authors:** Suresh Kumar, Nagarajan Perumal, P. K. Yadav, Ramendra Pati Pandey, Chung-Ming Chang, V. Samuel Raj

**Affiliations:** 1 National Institute of Biologicals, Ministry of Health & Family Welfare, Govt. of India, Noida, Uttar Pradesh 201309 India; 2 National Institute of Immunology, New Delhi, 110067 India; 3 All India Institute of Medical Sciences, New Delhi, 110029 India; 4 Center for Drug, Design, Discovery and Development (C4D), SRM University, Delhi-NCR, Sonepat, Haryana 131029 India; 5 Master & Ph.D. Program in Biotechnology Industry, Chang Gung University, No. 259, Wenhua 1st Rd., Guishan Dist., Taoyuan City, 33302 Taiwan, ROC

**Keywords:** Microbiology, Health care

## Abstract

Current evidence emerging from both human and animal models confirms that high-salt diet consumption over a period modulates the gut ecology and subsequently accelerates the development of the pathophysiology of many metabolic diseases. The knowledge of short-term intake of a high-salt diet (HSD) on gut microbiota and their role in the progression of metabolic pathogenesis and the consequence of a typical course of common antibiotics in this condition has yet not been investigated. The present study elicited this knowledge gap by studying how the gut microbiota profile changes in mice receiving HSD for a short period followed by Amoxicillin treatment on these mice in the last week to mimic a typical treatment course of antibiotics. In this study, we provided a standard chow diet (CD) and HSD for 3 weeks, and a subset of these mice on both diets received antibiotic therapy with Amoxicillin in the 3rd week. We measured the body weight of mice for 3 weeks. After 21 days, all animals were euthanised and subjected to a thorough examination for haemato-biochemical, histopathological, and 16S rRNA sequencing, followed by bioinformatics analysis to determine any changes in gut microbiota ecology. HSD exposure in mice for short duration even leads to a significant difference in the gut ecology with enrichment of specific gut microbiota crucially linked to developing the pathophysiological features of metabolic disease-related inflammation. In addition, HSD treatment showed a negative impact on haemato-biochemical parameters. However, Amoxicillin treatment in HSD-fed mice restored the blood-biochemical markers near to control values and reshaped gut microbiota known for improving the pathophysiological attributes of metabolic disease related inflammation. This study also observed minimal and insignificant pathological changes in the heart, liver, and kidney in HSD-fed mice.

## Introduction

Globally, most people nowadays consume too much salt in their diets unknowingly from processed foods and even consciously because of its pleasant taste^1,2^. A WHO report advised reducing salt intake to 5 g/day (2 g/day sodium) as most people consume more salt (9–12 g per day) than the recommended limit and reducing salt intake to this limit could prevent about 2.5 million deaths annually^1^. Animal studies revealed that an HSD can cause kidney damage via reactive oxygen species-dependent mechanisms. In addition, a high-salt diet also contributes to liver diseases like fibrosis and fatty liver through various mechanisms. Moreover, hypertension caused by HSD can also induce cardiovascular abnormalities by directly affecting endothelial functions and controlling vascular tone^3^. The gut microbiota drives the metabolism of the host by the interplay of multiple mechanisms like the production of bioactive metabolites, fermentation, and even indirectly by modulating immune responses^4^. The main bioactive metabolites such as bile acids, amino acids, and peptides are produced by the gut microbiota in response to the circulating signalling molecules in the host^5^. Trimethylamine-n-oxide (TMAO) is a significant product of the gut microbiota, which induces the expression of scavenger receptors that inhibit reverse cholesterol transport and accumulate cholesterol in macrophages, which accelerates the pathogenesis of atherosclerosis and other coronary artery diseases such as hypertension. Short-chain fatty acids (SCFAs) are a major metabolites of gut microbiota that interact with the olfactory receptor-78 (Olfr78) and the G Protein-Coupled Receptor 4 (GPR4) to regulate the host's blood pressure. Heart failure is usually linked with bacterially derived secondary bile acids and indoxyl sulphate^6^. Eating high salt over a period leads to a disruption in the microbial ecosystem homeostasis that increases the risk of multiple non-communicable diseases, especially chronic renal disorder, cardiovascular diseases, metabolic disorders, immune-related disorders, and premature death^7–10^. Animal studies have displayed that a high-salt diet harms gut health by triggering systemic inflammatory conditions resulting in damage to target organs^11^. Researchers have highlighted the crucial role of the gut microbiota in contributing to human health and diseases. Now, attention is turning towards intestinal microflora and related metabolites as potential therapeutic targets. Usually, antibiotics are lifesaving therapeutic drugs used to eradicate bacterial pathogens that also induce a change in the intestinal microbiota, disrupting the host immune and nutritional homeostasis and decreasing colonisation resistance^12,13^. Another study in mice proved that antibiotics cause modulation of gut bacteria and associated circulating LPS levels in obesity by improving insulin tolerance and glucose tolerance in metabolically active tissues^14^. However, there is growing interest to know as how antibiotics treatment impacts in the backdrop of HSD diet may influence on gut bacteria, haemato-biochemical parameters, and histopathological changes in vital organs. This understanding is highly pragmatic now as the modern diet has higher salt than the recommended level, and antibiotics are commonly given during infections to treat patients. Notwithstanding how antibiotics likely influence the resident gut microbiota by improving or deteriorating the pathophysiology of individuals on an HSD diet, it remains poorly explored. We performed this study to address these gaps by conducting experiments, wherein the mice were fed an HSD diet for 3 weeks and provided Amoxicillin treatment in the 3rd week to follow a typical course of antibiotics. We selected Amoxicillin antibiotic in this study because the primary healthcare settings used it as the most common antibiotic^15^. In this investigation, we first showed how short-term intake of HSD for 3 weeks caused change in the overall community structure of the gut microbiota, Afterwards, we examined how the short course of Amoxicillin treatment reshaped the gut microbiota by employing 16S rRNA high-throughput sequencing. We also projected the haematological, biochemical profile changes and histopathological effects on vital organs, namely the heart, liver, and kidney in the mouse model.

## Methods

### Animal model

Twenty-four male C57BL/6 J mice aged 6–8 weeks were kept under standard environmental conditions (22–24 °C, 12 h light/dark cycles) with free access to water and chow at All India Institute of Medical Sciences (AIIMS), New Delhi. All experimental procedures complied with the standards stated in the ARRIVE guidelines and Committee for the Purpose of Control and Supervision of Experiments on Animals (CPCSEA) and were conducted under conditions approved by the Institutional Animal Ethics Committee (IAEC) of AIIMS. All methods were performed in accordance with the relevant guidelines.

### Study design

Mice from the same cohort were randomly divided into two groups (N = 12 for each group) and fed CD (0.4% NaCl in chow) and HSD (4% NaCl in chow) for 3 weeks. Half of the animals (N = 6) in each group were given Amoxicillin treatment in the 3rd week at a 50 mg/kg body weight dose, i.e., 0.25 mg/ml in drinking water. The dosage and concentration of Amoxicillin in drinking water was based on published literature^16^. The treatment/diet group was referred to as CD + Amox as a positive control and HSD + Amox for treated mice. Another half of the animals (N = 6) from each group did not receive Amoxicillin treatment. These diet groups were designated CD for control and HSD for treated mice. The weight of mice was measured weekly and analysed once a week. After 3 weeks, these animals were euthanized to collect samples to see the impact of Amoxicillin on HSD and CD groups.

### Blood, tissue, and caecal sample collection

After 3 weeks, all animals overnight fasted, and blood samples were taken by cardiac puncture. Then the vital organs, namely the heart, liver, and kidney, were harvested for histopathological examination. Caecal content samples were taken immediately after dissection of mice and frozen in liquid nitrogen at − 80 °C until further 16S rRNA gene sequencing was performed.

### Serum biochemistry and haematology

Blood biochemistry was assessed using the serum auto-analyzer Screen Master 3000, Tulip, Alto Santa Cruz, India, with the Coral GPO-PAP kit (CORAL Clinical systems, Goa, India) according to manufacturer's directions. The haematological analysis was conducted using an automated vet haematology counter (Melet Schloesing Laboratories, Guwahati, India) as per the manufacturer’s usage guide.

### Histopathology

Vital organ tissues, namely heart, liver, kidney were fixed overnight in 10% neutral buffered formalin. Tissues embedded in paraffin blocks were cut into 4–5 μm thick sections using a microtome. According to standard protocols, tissue sections were subjected to deparaffinization, rehydration, and haematoxylin and eosin (H&E) staining. The digital images were taken by light microscopy (Olympus CX-29: Olympus Optical Co. Ltd, Tokyo, Japan) and camera (Magnus DC 10).

### High throughput 16S rRNA gene amplicon sequencing

According to the manufacturer's instructions, the total genomic DNA of gut microbiota was extracted from caecal content samples by using Qiagen DNA Stool Mini Kit. The samples were sent for high throughput 16S rRNA gene amplicon sequencing and genus analysis (DNA Xperts Private Limited, India). After DNA extraction, the samples were expanded by using the universal 16sPCR primer specific for the V3–V4 region included: 341F 5′- CCTAYGGGRBGCASCAG-3′ and 806R 5′-GGACTACNNGGGTATCTAAT-3′ according to the Illumina Miseq high-throughput sequencer usage guide.

### Microbial bioinformatics analysis

Sequence data was analysed using QIIME (v1.9.1) software. FastQC and Trimmomatic were applied to trim and align the paired-end reads with tags with an average read length of 252 bp^17^. The tags were then clustered as OTUs (Operational Taxonomic Units) with a 97% similarity threshold^18^. Rarefaction curves were used to ensure the sufficient sequencing depth of samples. The relative abundance of each sample was calculated based on the normalised operational taxonomic units (OTUs). Alpha diversity was estimated by species richness (Chao) and diversity index (Shannon, Simpson). Beta diversity among the group samples was calculated mainly using the unweighted and weighted UniFrac distances, Bray–Curtis, and Jaccard distances. Principal coordinate analysis (PCoA) distance matrices created by QIIME^19^.

### Statistical analysis

GraphPad Prism version 9.2.0 (3.2.0) for Windows, Graph Pad Software, San Diego, California USA, www.graphpad.com" was used for the statistical analysis of experimental data. Values are expressed as the means ± Standard Deviation (SD). Groups were compared by one-way analysis of variance (ANOVA) followed by the Bonferroni test for comparisons between multiple groups with a value of *p* ≤ 0.05 as the cut-off for statistical significance.

## Results

### Short term HSD treatment and Amoxicillin treatment to HSD-fed mice did not cause significant difference in weight of mice

**Figure 1:**
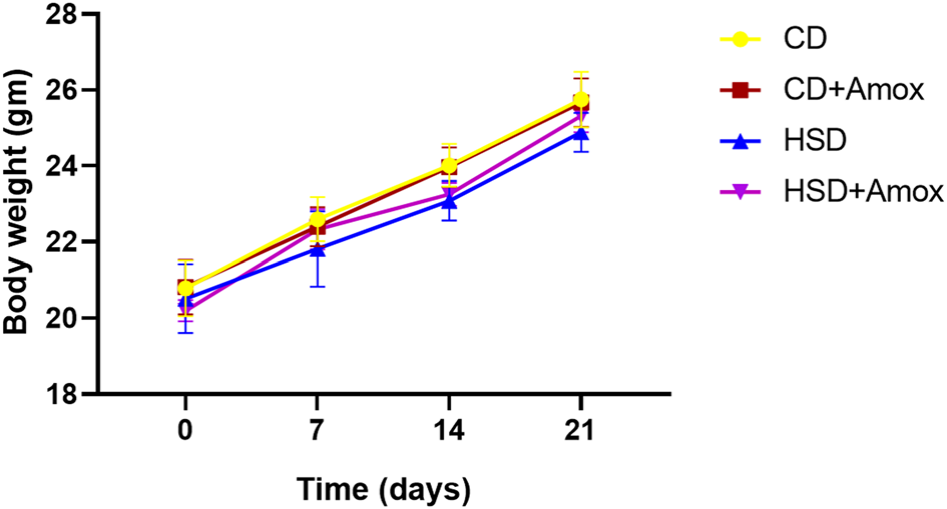
Effect of high-salt diet (4% in chow) and Amoxicillin treatment on body weight of mice. As shown by the data, no significant difference in body weight of mice was observed among different groups. Data represent mean ± SD; N = 6 mice/group, One Way ANOVA (*p < 0.05, **p < 0.01, ***p < 0.001. CD: Standard—chow diet for 3 weeks; CD + Amox: Standard—chow diet for 3 weeks and subjected to Amoxicillin treatment in 3rd week; HSD: High-salt diet for 3 weeks; HSD + Amox: High-salt diet for 3 weeks and subjected to Amoxicillin treatment in 3rd week.

In this study, HSD-fed mice did not display a significant difference in weight when compared to CD-fed mice (Fig. 1).. Likewise, we also did not observe a significant weight difference in HSD-fed mice and Amoxicillin treated HSD-fed mice (Fig. 1).. Thus, the present investigation indicates that a high salt diet intake for a short period could not lead to weight gain in mice.

### Amoxicillin treatment restores the HSD-promoted thrombocytosis

**Figure 2:**
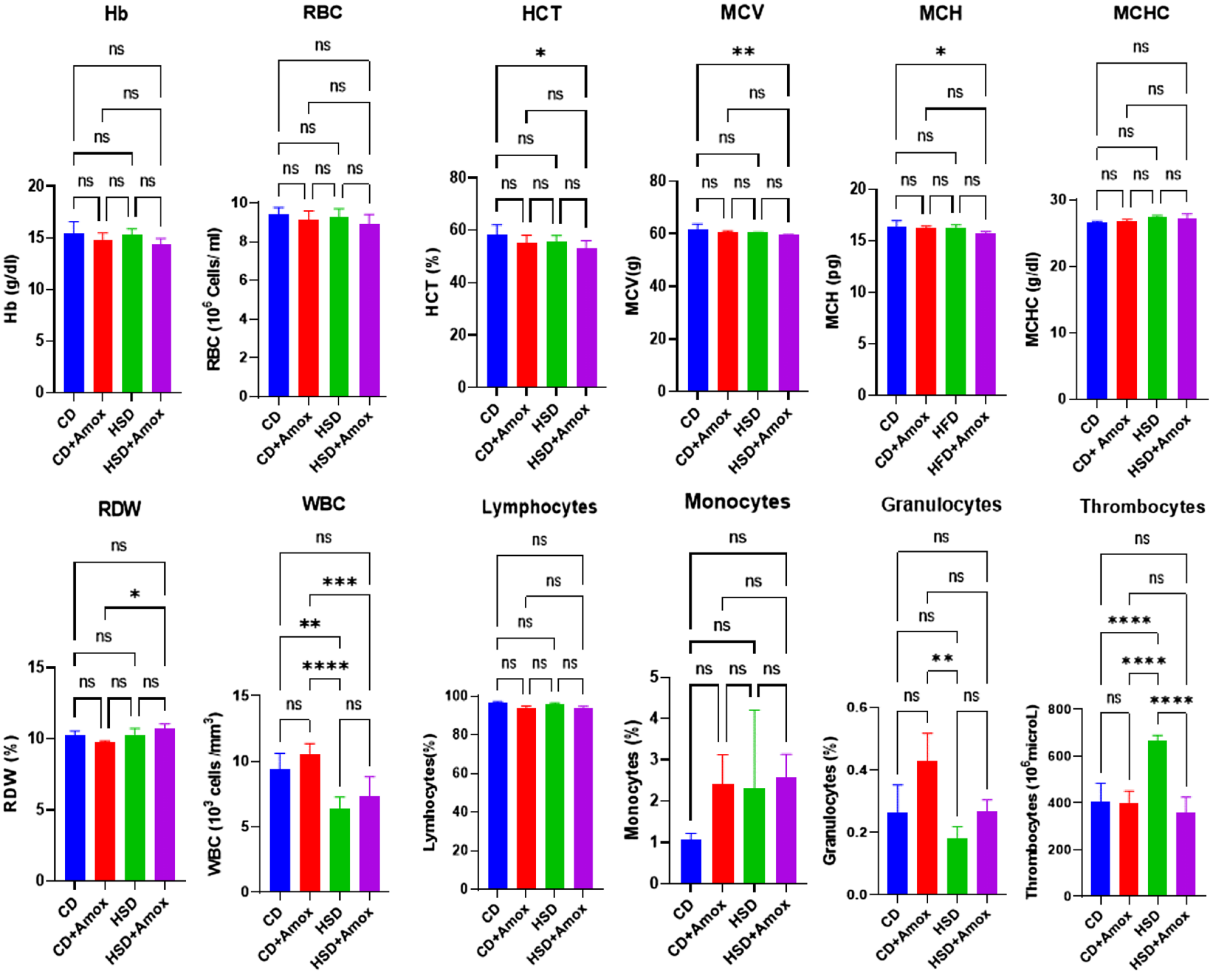
Short-term effect of high-salt diet (4% in chow) followed by Amoxicillin treatment on haematological parameters in mice. HSD treatment for 3 weeks in mice increases the thrombocytes concentration. Amoxicillin treatment in the 3rd week in HSD fed mice brought the elevated level of the thrombocytes near to control value. Values are means ± SD for 6 samples in each group. Statistically significance of differences was evaluated by one way ANOVA followed by Bonferroni test (*p < 0.05, **p < 0.01, ****p < 0.0001). CD: Standard—chow diet for 3 weeks; CD + Amox: Standard—chow diet for 3 weeks and subjected to Amoxicillin treatment in 3rd week; HSD: High-salt diet for 3 weeks; HSD + Amox: High-salt diet for 3 weeks and subjected to Amoxicillin treatment in 3rd week.

This study first examined the impact of Amoxicillin treatment in HSD-fed mice on haematological parameters, as shown in Fig. 2 and Table 1. We observed a significant decrease of white blood cells (WBCs) in the HSD-fed mice compared to the CD-fed mice group (*p* = 0.0016). But the Amoxicillin treatment did not find any significant alteration in WBC values as observed in the HSD + Amox group in comparison to the HSD group. However, we found a significant increase in the thrombocytes in the HSD group in comparison to the CD group (*p* < 0.0001). Interestingly, in HSD-fed mice treated with Amoxicillin showed a significant decrease in the thrombocytes compared with their HSD-fed counterparts (*p* < 0.0001). No significant alteration of haematological parameters in the CD + Amox group compared to the CD group was distinguished. Overall, our study also suggests that Amoxicillin treatment could likely restore thrombocyte value to normal range in the pathophysiology associated with HSD. However, Amoxicillin therapy did not impact the value of thrombocytes in the CD fed mice.

**Table 1:** Short term effect high salt (4% in chow) diet followed by Amoxicillin treatment on hepatological parameters in mice.

Parameters	Groups				Significance (*p*-value)		
	CD	CD + A	HSD	HSD + A	CD versus CD + A	CD versus HSD	HSD versus HSD + A
Hb (g/dl)	15.46 ± 1.1	14.83 ± 0.6	15.33 ± 0.5	14.43 ± 0.5	NS	NS	NS
RBC (10^6^ cells/ml)	9.40 ± 0.3	9.1 ± 0.4	9.26 ± 0.4	8.91 ± 0.4	NS	NS	NS
HCT (%)	58.23 ± 3.8	55.23 ± 2.8	55.43 ± 2.4	53.0 ± 2.9	NS	NS	NS
MCV (g)	61.9 ± 1.8	60.76 ± 0.3	60.5 ± 0.1	59.53 ± 0.2	NS	NS	NS
MCH (pg)	16.36 ± 0.5	16.26 ± 0.1	16.5 ± 0.2	16.16 ± 0.4	NS	NS	NS
MCHC(g/dl)	26.5 ± 0.2	26.83 ± 0.2	27.3 ± 0.2	27.2 + 0.7	NS	NS	NS
RDW (%)	10.25 ± .0.34	9.72 ± 0.11	10.2 ± 0.52	10.7 ± 0.30	NS	NS	NS
WBC (10^6^ cells/ml)	9.41 ± 1.1	10.52 ± 0.8	6.45 ± 0.8	7.35 ± 1.4	NS	0.0016	NS
Lymphocytes (10^6^ cells/ml)	96.3 ± 0.7	93.6 ± 1.3	95.96 ± 0.5	93.8 ± 0.9	NS	NS	NS
Monocytes (%)	1.06 ± 0.1	2.4 ± 0.7	2.3 ± 1.9	2.56 ± 0.5	NS	NS	NS
Granulocytes (%)	0.26 ± 0.08	0.43 ± 0.08	0.18 ± 0.03	0.27 ± 0.03	NS	NS	NS
Thrombocytes (10^3^/microL)	407.66 ± 76.2	400.3 ± 50.5	664 ± 25.2	356.66 ± 67.9	NS	< 0.0001	< 0.0001

Values are expressed as the means ± SD. Data were analyzed by one-way ANOVA, followed by Bonferroni test, n = 6, *p* ≤ 0.05, NS = Not significant, CD: Standard—chow diet for 3 weeks; CD + A: Standard—chow diet for 3 weeks and subjected Amoxicillin treatment in 3rd week; HSD: High-salt diet for 3 weeks; HSD + A: High-salt diet for 3 weeks and subjected to Amoxicillin treatment in 3rd week.

### Amoxicillin treatment declines the HSD-promoted blood glucose level

**Figure 3:**
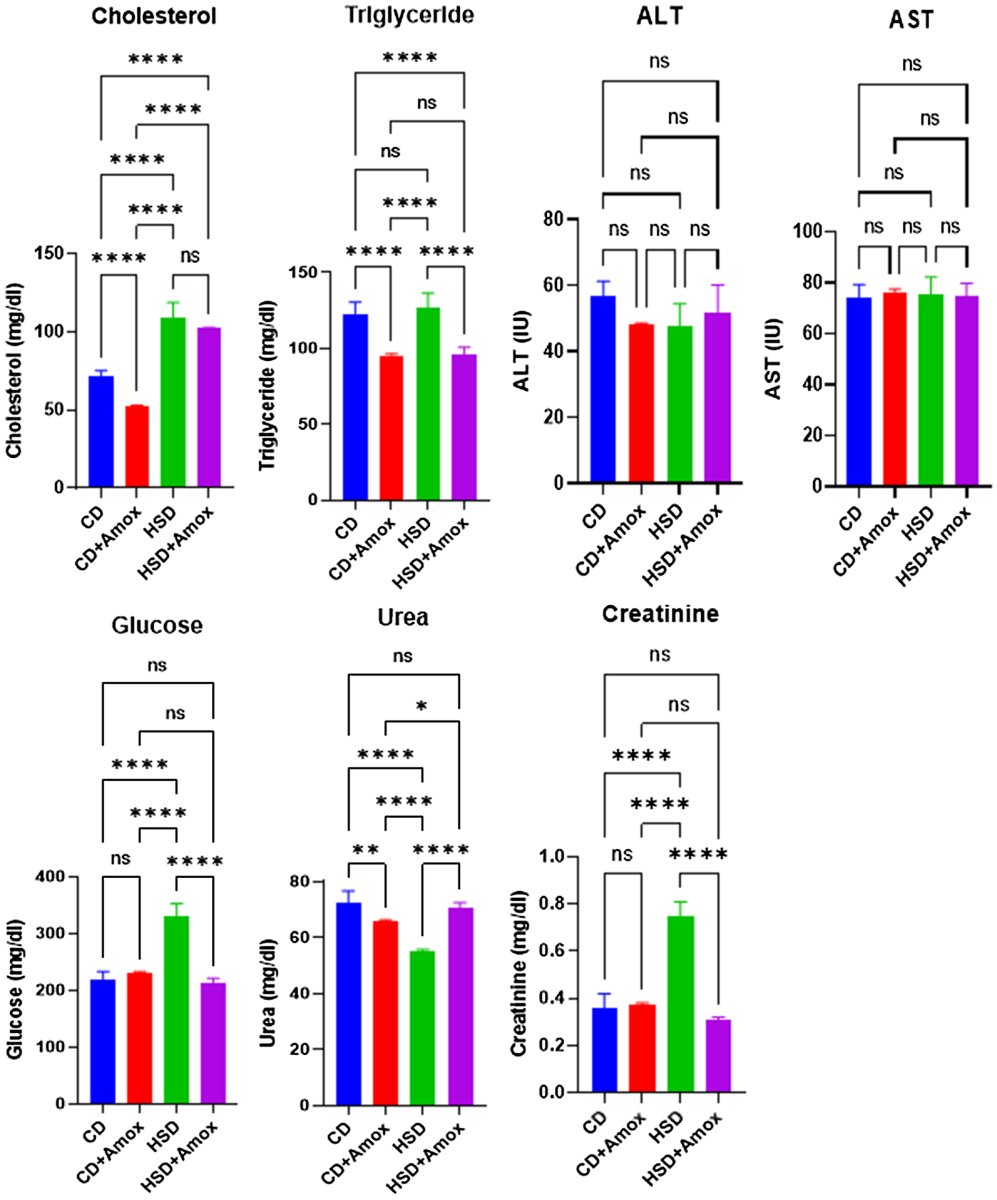
Short-term effect of high-salt diet (4% in chow) followed by Amoxicillin treatment on biochemical parameters in mice. HSD treatment for 3 weeks in mice showed a significant increase in total cholesterol, glucose, creatinine and a significant decrease in blood urea level. Amoxicillin treatment in the last one week in HSD fed mice caused restoration of the glucose, triglycerides, creatinine and blood urea levels towards the control value. Values are means ± SD for 6 samples in each group. Statistical significance of differences was evaluated by one way ANOVA followed by Bonferroni test (*p < 0.05, **p < 0.01, ***p < 0.001, ****p < 0.0001). CD: Standard—chow diet for 3 weeks; CD + Amox: Standard—chow diet for 3 weeks and subjected to Amoxicillin treatment in 3rd week; HSD: High-salt diet for 3 weeks; HSD + Amox: High-salt diet for 3 weeks and subjected to Amoxicillin treatment in 3rd week.

Next, we demonstrate the effect of Amoxicillin in HSD-fed mice on biochemical parameters involved in metabolic syndrome (Fig. 3 and Table 2). Our data showed a significant increase in blood glucose and total cholesterol (*p* < 0.0001) in HSD-fed mice compared to the CD-fed mice. Amoxicillin treatment in HSD-fed mice significantly reduced glucose levels (*p* < 0.0001) but found no significant change in cholesterol levels. Amoxicillin treatment in the standard chow diet group showed no alteration in cholesterol and glucose level. The results of the study suggest that Amoxicillin treatment could likely restore blood glucose levels towards normal range in the HSD induced pathophysiology.

**Table 2:** Short term effect high salt (4% in chow) diet followed by Amoxicillin treatment on biochemical parameters in mice.

Parameters	Groups				Significance (*p*-value*)*		
	CD	CD + A	HSD	HSD + A	CD versus CD + A	CD versus HSD	HFD versus HSD + A
Cholesterol (mg/dl)	71.49 ± 3.6	52.7 ± 0.3	108.91 ± 9.8	102.34 ± 0.3	< 0.0001	< 0.0001	NS
Triglycerides(mg/dl)	122.99 ± 7.5	95.44 ± 0.9	127.21 ± 9.4	95.78 ± 5.0	< 0.0001	NS	< 0.0001
ALT (IU)	56.80 ± 4.5	48.04 ± 0.5	47.86 ± 6.5	51.49 ± 8.6	NS	NS	NS
AST (IU)	74.47 ± 4.8	75.89 ± 1.6	75.19 ± 7.08	74.81 ± 4.9	NS	NS	NS
Creatinine (mg/dl)	0.36 ± 0.06	0.37 ± 0.01	0.75 ± 0.06	0.31 ± 0.01	NS	< 0.0001	< 0.0001
Urea (mg/dl)	72.21 ± 4.5	66.15 ± 0.2	54.99 ± 0.8	70.88 ± 1.6	0.0029	< 0.0001	< 0.0001
Glucose (mg/dl)	219.8 ± 13.08	231.54 ± 0.89	329.2 ± 23.6	213.46 ± 7.2	NS	< 0.0001	< 0.0001

Values are expressed as the means ± SD. Data were analyzed by one-way ANOVA, followed by Bonferroni test, n = 6, *p* ≤ 0.05, NS = Not significant, CD: Standard—chow diet for 3 weeks; CD + A: Standard—chow diet for 3 weeks and subjected to Amoxicillin treatment in 3rd week; HSD: High-salt diet for 3 weeks; HSD + A: High-salt diet for 3 weeks and subjected to Amoxicillin treatment in 3rd week.

### Amoxicillin treatment decreases the HSD-promoted blood creatinine level

We then examined the effect of Amoxicillin treatment on blood creatinine level in HSD-fed mice (Fig. 3 and Table 2). Our data observed a significant increase in creatinine in HSD-fed mice compared to CD-fed mice (*p* < 0.0001). However, Amoxicillin-treated HSD-fed mice showed a significant reduction in creatinine (*p* < 0.0001) level compared to HSD-fed mice. There was no significant difference in creatinine level between the Amoxicillin-treated CD-fed mice and the CD-fed mice. Our study suggests that Amoxicillin treatment in HSD-fed mice could likely decrease the creatinine towards the normal range.

### Amoxicillin treatment restores the HSD-decreased blood urea level

In addition, we investigated the effect of Amoxicillin on blood urea level in HSD-fed mice (Fig. 3 and Table 2). Our data showed a significantly reduced urea level in HSD-fed mice compared to CD-fed mice (*p* < 0.0001). Amoxicillin treatment in HSD-fed mice substantially increases the urea concentration compared to HSD-fed mice (*p* < 0.0001). However, Amoxicillin-treated CD-fed mice also showed a significant decrease in urea concentration (*p* = 0.0029). Our data suggest that Amoxicillin treatment in HSD-fed could restore urea level to a normal range.

### Amoxicillin treatment depletes HSD induced richness and diversity of especially of different gut bacteria

**Figure 4:**
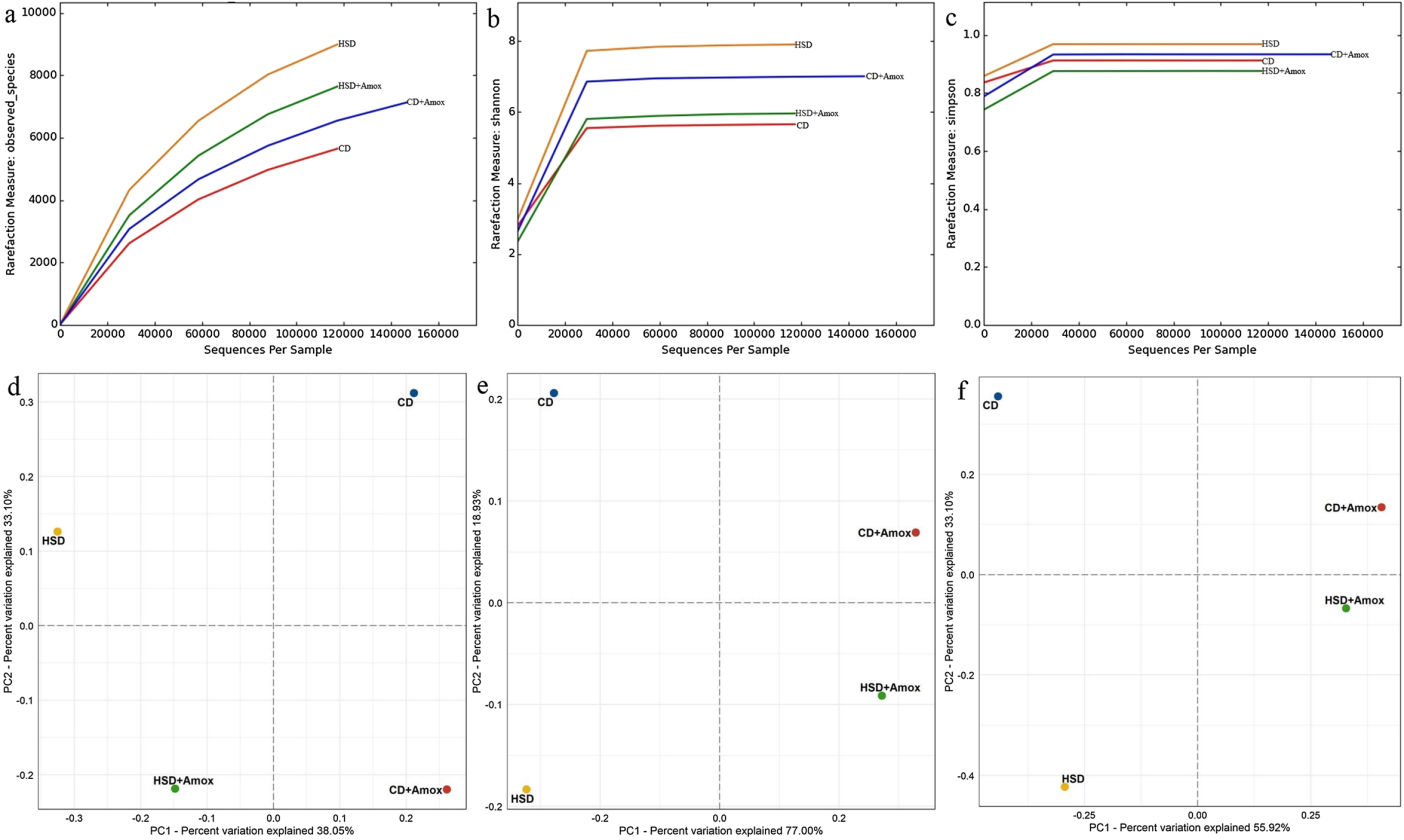
Short-term effect of high-salt diet (4% in chow) followed by Amoxicillin treatment on gut bacteria diversity in mice. Rarefaction curves (**a**) and Shannon–Wiener curves (**b**) achieved a plateau, suggesting that the number of OTUs was sufficient to capture the authentic bacterial communities in each sample. Comparisons for alpha-diversity such as observed species index (**a**), Shannon (**b**) and Simpson (**c**) index and beta-diversity (with unweighted (**d**), weighted (**e**) Unifrac distance matrix and Bray–Curtis dissimilarity matrix (**e**) showed marked differences in the bacterial communities among the groups. The percent variation explained by each group is indicated on the axis. CD: Standard—chow diet for 3 weeks; CD + Amox: Standard—chow diet for 3 weeks and subjected to Amoxicillin treatment in 3rd week; HSD: High-salt diet for 3 weeks; HSD + Amox: High-salt diet for 3 weeks and subjected to Amoxicillin treatment in 3rd week.

After initial quality control filtering, we generated a dataset of 1,169,822 high-quality sequences with an average of 292,455 reads per group sample. The high-quality sequences were clustered with a consistency of 97% similarity level and obtained a total number of 22,980 OTUs, as shown in Table 3. The Shannon–Wiener curve of all four samples already reached a plateau (Fig. 4), suggesting that the sequencing was deep enough to capture the entire spectrum of bacterial diversity and the majority of information pertaining to dominant phylotypes in each group. Estimators of the alpha and beta diversity are summarised in Tables 3 and 4 and shown in Fig. 4. Next, we calculated and compared the Chao1, Shannon, and Simpson indices of alpha diversity and observed species as outlined in Table 4 for each group. We found significantly higher alpha diversity estimators like Chao1 (richness) and Shannon (diversity of rare species) in the HSD group than in the Amoxicillin-treated HSD and CD-fed mice. The alpha diversity indices illustrated that HSD increased the richness and diversity of organisms, especially different gut bacteria. Amoxicillin treatment in HSD-fed mice showed a reduction in richness and diversity, especially of specific gut bacteria. There was no statistically significant difference for the Simpson index, indicating a remarkable similarity in general microbial diversity in all four group samples. Beta diversity measures of microbiota (shown in Table 4 and Fig. 4) based on principal coordinates analysis (PCoA) of weighted Unifrac distance and Bray–Curtis dissimilarity showed a distinguished separation among four groups, suggesting that the composition of microbiota was significantly distinct from each other.

**Table 3:** Short term effect of high-salt (4% in chow) diet followed by Amoxicillin treatment on alpha diversity.

Alpha diversity indices	Groups				Significance (*p-*value)		
	CD	CD + A	HSD	HSD + A	CD versus CD + A	CD versus HSD	HFD versus HSD + A
OTUs	6468.7 ± 568.6	4871.6 ± 678.5	7844.45 ± 1004.47	6606.76 ± 905.07	0.0013	0.0331	NS
Chao1	8682.7 ± 42.69	7152.3 ± 74.4	10,901.16 ± 150.73	9541.055 ± 156.6	< 0.0001	< 0.0001	< 0.0001
Shannon	6.99 ± 0.0132	5.63 ± 0.020	7.86 ± 0.029	5.93 ± 0.028	< 0.0001	< 0.0001	< 0.0001
Simpson	0.933 ± 1.110	0.912 ± 0.00	0.968 ± 0.000	0.867 ± 0.000	NS	NS	0.0371

Values are expressed as the means ± SD. Data were analyzed by one-way ANOVA, followed by Bonferroni test, n = 6, *p* ≤ 0.05, NS = Not significant, CD: Standard—chow diet for 3 weeks; CD + A.: Standard—chow diet for 3 weeks and underwent Amoxicillin treatment in 3rd week; HSD: High-salt diet for 3 weeks; HFD + A.: High-salt diet for 3 weeks and underwent Amoxicillin treatment in 3rd week.

**Table 4:** Short term effect of high-salt diet (4% in chow) followed by Amoxicillin treatment on beta diversity.

Beta diversity indices	Groups (R-value)		
	CD v s CD + A	CD versus HSD	HSD versus HSD + A
unweighted UniFrac distance	0.633809644	0.662522256	0.622666243
weighted UniFrac distance	0.632525949	0.399167807	0.616484456
Bray–Curtis dissimilarity	0.909346046	0.799767498	0.782961221
Jaccard Abundance	0.201126632	0.226449915	0.193867315

CD: Standard—chow diet for 3 weeks; CD: Standard—chow diet for 2 weeks; CD + A.: Standard—chow diet for 3 weeks and underwent Amoxicillin treatment last week; HSD: High-salt diet for 3 weeks; HFD + A.: High-salt diet for 3 weeks and underwent Amoxicillin treatment last week.

### Amoxicillin treatment reshapes the specific gut microbial community associated with the improvement in the pathophysiology of metabolic syndrome and increases the risk of some opportunistic gut pathogens

**Figure 5:**
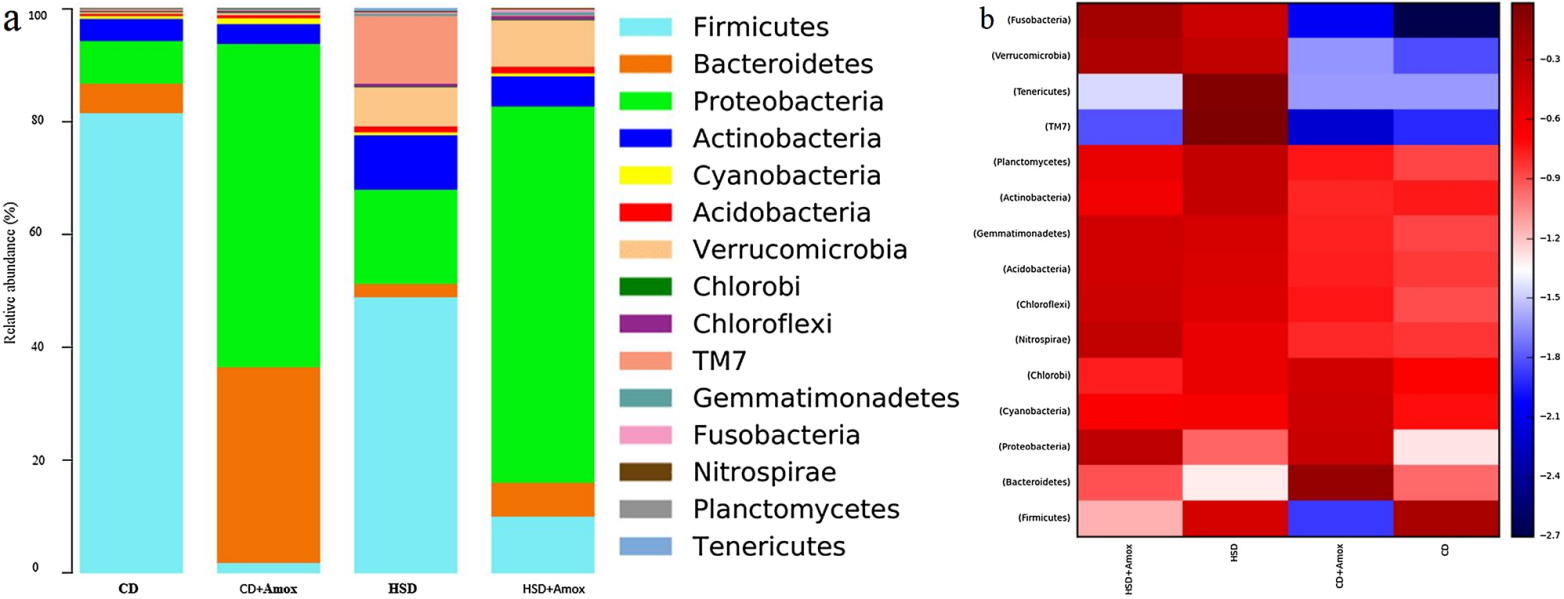
Bar chart (**a**) and heat map (**b**) showed the short-term effect of a high-salt diet (4% in chow) followed by Amoxicillin treatment on the relative abundance of the most represented phylum for each group. HSD treatment for 3 weeks in mice caused a significant increase of *Firmicutes* to *Bacteroidetes* (*F/B*) ratio and *Proteobacteria*. During the last week, Amoxicillin treatment in HSD fed mice mice caused a significant decrease in the *Firmicutes* to *Bacteroidetes* (*F/B*) ratio and as well as increased of *Proteobacteria*. Color heat map (4b) analysis illustrating the top 15 mean abundances of the bacterial community taxa assigned to phyla. The color intensity in each sample is normalized to represent its relative ratio in the four groups. The color scale of higher (dark red) and lower (dark blue) shows the relative abundances of bacterial communities. CD: Standard—chow diet for 3 weeks; CD + Amox: Standard—chow diet for 3 weeks and subjected to Amoxicillin treatment in 3rd week; HSD: High-salt diet for 3 weeks; HSD + Amox: High-salt diet for 3 weeks and subjected to Amoxicillin treatment in 3rd week.

Next, we performed pairwise differential alteration analysis on gut microbiota to identify specific gut bacteria with potential clinical importance in the pathophysiology of metabolic syndrome and the subsequent impact of Amoxicillin on the modulation of these gut microbiota. We conducted core bacteria analysis at the phylum level (Table 5, Fig. 5) that demonstrates a significant decrease in *Firmicutes* (*p* = 0.0028), *Bacteroidetes* (*p* < 0.0001) and an increase of *Proteobactera* (*p* < 0.0001) in HSD-fed mice compared to standard CD-fed mice. However, Amoxicillin treatment for one week in HSD-fed mice significantly further decreased *Firmicutes* (*p* = 0.0009) and increased *Bacteroidetes* (*p* < 0.0001). Similarly, Amoxicillin treatment in CD-fed mice also showed a decrease in *Firmicutes* (*p* < 0.0001) considerably and increase in *Bacteroidetes* (*p* < 0.0001). The relative abundance of *Proteobacteria* increased dramatically in HSD-fed mice compared to CD-fed mice (*p* < 0.0001). Amoxicillin-treated HSD-fed mice showed a significant increase in the abundance of *Proteobacteria* (*p* < 0.0001). Similarly, Amoxicillin treatment in CD-fed mice caused a significant increase in the abundance of *Proteobacteria* (*p* < 0.0001). The *Firmicutes/ Bacteroidetes* (*F/B*) ratio was significantly higher in HSD-fed as compared to mice CD-fed mice Amoxicillin treatment in HSD-fed mice substantially reduces the *F/B* ratio compared to HSD *(p* < 0.0001). Similarly, Amoxicillin-treated CD mice showed a significant decrease in the *F/B* ratio (*p* < 0.0001).

**Table 5:** Short term effect of high salt (4% in chow) diet followed by Amoxicillin treatment on the mean relative abundance of major bacterial groups at phylum level in mice.

Phylum	Relative abundance (%)				Significance (Adjusted *p*-value)		
	CD	CD + A	HSD	HSD + A	CD versus CD + A	CD versus HSD	HSD versus HSD + A
*Firmicutes*	80.30 ± 0.397	1.80 ± 0.132	47.40 ± 0.499	9.80 ± 0.297	< 0.0001	0.0028	0.0009
*Bacteroidetes*	5.10 ± 0.219	56.30 ± 0.496	2.30 ± 0.149	5.90 ± 0.235	< 0.0001	< 0.0001	< 0.0001
*Proteobacteria*	7.50 ± 0.264	34.00 ± 0.473	16.20 ± 0.368	65.3 ± 0.467	< 0.0001	< 0.0001	< 0.0001
*Actinobacteria*	3.90 ± 0.193	3.60 ± 0.186	9.30 ± 0.290	5.20 ± 0.022	NS	< 0.0001	< 0.0001
*Cyanobacteria*	0.50 ± 0.070	0.90 ± 0.094	0.60 ± 0.077	0.60 ± 0.077	< 0.0001	0.0159	NS
*Acidobacteria*	0.40 ± 0.063	0.50 ± 0.070	1.00 ± 0.099	1.10 ± 0.104	0.0264	< 0.0001	< 0.0001
*Verrucomicrobia*	0.20 ± 0.044	0.40 ± 0.063	6.70 ± 0.250	8.10 ± 0.272	< 0.0001	< 0.0001	< 0.0001
*Chlorobi*	0.10 ± 0.031	0.20 ± 0.044	0.10 ± 0.031	0.10 ± 0.031	0.0011	NS	NS
*Chloroflexi*	0.20 ± 0.044	0.30 ± 0.054	0.50 ± 0.070	0.60 ± 0.077	0.0056	< 0.0001	NS
*TM7*	0.10 ± 0.031	0.10 ± 0.031	11.60 ± 0.320	0.20 ± 0.044	NS	< 0.0001	< 0.0001
*Gemmatimonadetes*	0.20 ± 0.044	0.20 ± 0.044	0.50 ± 0.070	0.50 ± 0.070	NS	< 0.0001	NS
*Fusobacteria*	0.00 ± 0.000	0.00 ± 0.000	0.30 ± 0.054	0.40 ± 0.063	NS	< 0.0001	0.0076
*Nitrospirae*	0.10 ± 0.031	0.10 ± 0.031	0.20 ± 0.044	0.30 ± 0.054	NS	0.0040	0.0040
*Planctomycetes*	0.00 ± 0.000	0.00 ± 0.000	0.10 ± 0.031	0.00 ± 0.000	NS	< 0.0001	< 0.0001
*Tenericutes*	0.00 ± 0.000	0.00 ± 0.000	0.50 ± 0.070	0.00 ± 0.000	NS	< 0.0001	< 0.0001
*F/B ratio*	15.74 ± 0.363	0.05 ± 0.022	20.6 ± 0.404	1.66 ± 0.125	< 0.0001	< 0.0001	< 0.0001

Values are expressed as the means ± SD. Data were analyzed by one-way ANOVA, followed by Bonferroni test, n = 6, *p* ≤ 0.05, NS = Not significant, CD: Standard—chow diet for 3 weeks; CD + A: Standard—chow diet for 3 weeks and subjected to Amoxicillin treatment in 3rd week; HSD: High-salt diet for 3 weeks; HSD + A: High-salt diet for 3 weeks and subjected to Amoxicillin treatment in 3rd week.

**Figure 6:**
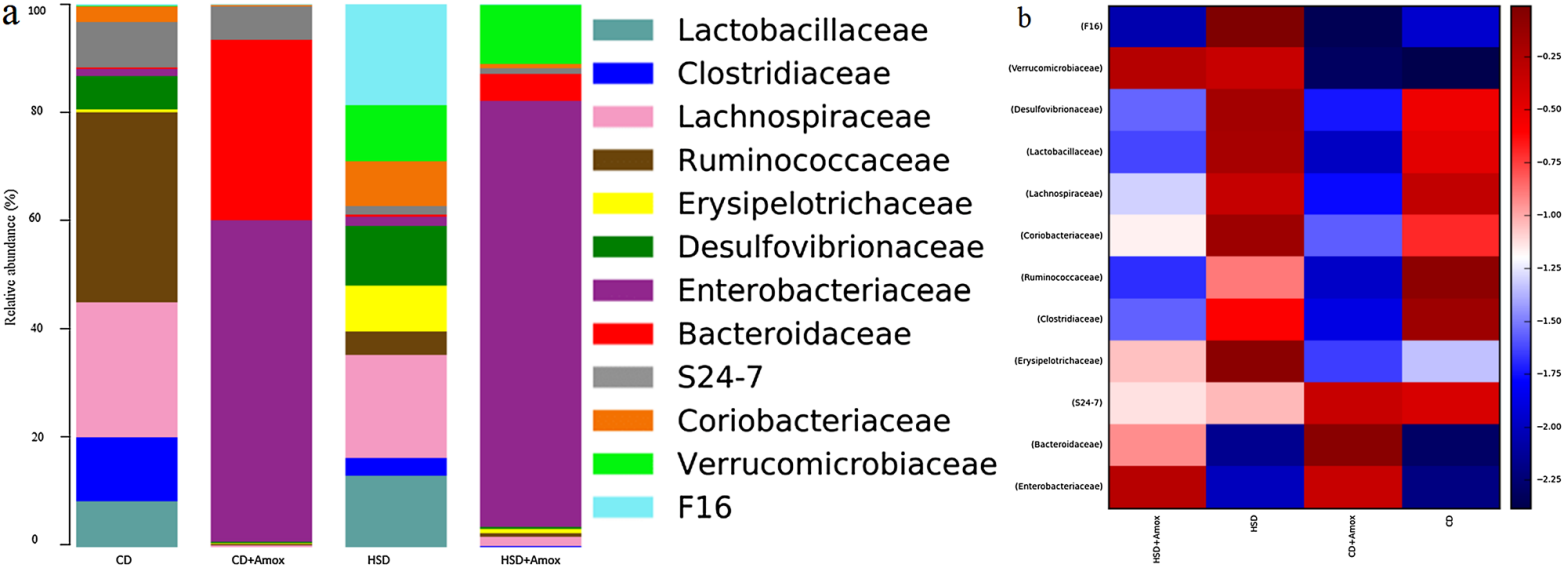
Bar chart (**a**) and heat map (**b**) showed the short-term effect of a high-salt diet (4% in chow) followed by Amoxicillin treatment on the relative abundance of the most represented family for each group. Amoxicillin-treatment in HSD-fed mice found a significant decrease in *Erysipelotrichaceae*, *Desulfovibrionaceae*, *Coriobacteriaceae*, *F16* and a significant increase in *Bacteroidaceae*, *Enterobacteriaceae* and *Verrucomicrobiaceae*. Color heat map (**b**) analysis illustrating the top 12 mean abundances of the bacterial community taxa assigned to family. The color intensity in each sample is normalized to represent its relative ratio in the four groups. The color scale of higher (dark red) and lower (dark blue) shows the relative abundances of bacterial communities. CD: Standard—chow diet for 3 weeks; CD + Amox: Standard—chow diet for 3 weeks and subjected to Amoxicillin treatment in 3rd week; HSD: High-salt diet for 3 weeks; HFD + Amox: High-salt diet for 3 weeks and subjected to Amoxicillin treatment in 3rd week.

We observed a total of 45, 53, 71, and 69 families (Table 6 and Fig. 6) in the CD, CD + Amox, HSD, and HSD + Amox group samples, respectively. There was a significant increase in the abundance of *Erysipelotrichiciae*, *Desulfovibrionaceae*, *Coriobacteriaceae* and *F16* (*p* < 0.0001) in HSD-fed mice. Amoxicillin-treated HSD-fed mice showed a significant decrease in *Erysipelotrichaceae*, *Desulfovibrionaceae*, *Coriobacteriaceae,* and *F16* in comparison to HSD-fed mice (*p* < 0.0001). Amoxicillin treatment in CD-fed mice also caused a significant decrease in the *Erysipelotrichaceae, Desulfovibrionaceae,* and *Coriobacteriaceae* compared to CD-fed mice (*p* < 0.0001). HSD-fed mice also showed significant increases in *Verrucomicrobiaceae* and *Enterobacteriaceae* (*p* < 0.0001) compared to CD-fed mice. Amoxicillin treatment in HSD-fed mice further significantly increased *Verrucomicrobiaceae* and *Enterobacteriaceae* (*p* < 0.0001). HSD-fed mice showed a significant increase in *Enterobacteriaceae* (*p* < 0.0001) but did not alter *Bacteroidaceae* in comparison to the CD-fed mice. Amoxicillin resentment caused a significant increase in *Enterobacteriaceae* and *Bacteroidaceae* (*p* < 0.0001). Similarly, Amoxicillin-treated CD-fed mice also showed a significant increase in the abundance of *Enterobacteriaceae* and *Bacteroidaceae* (*p* < 0.0001).

**Table 6:** Short term effect of high-salt (4% in chow) diet followed by amoxicillin treatment on the mean relative abundance of major bacterial groups at family level in mice.

Family	Relative abundance (%)				Significance (Adjusted *p*-value)		
	CD	CD + A	HSD	HSD. + A	CD versus CD + A	CD versus HSD	HSD versus HSD + A
*Lactobacillaceae*	4.50 ± 0.207	0.1 ± 0.031	8.50 ± 0.278	0.30 ± 0.054	< 0.0001	< 0.0001	< 0.0001
*Clostridiaceae*	5.90 ± 0.235	0.1 ± 0.031	2.10 ± 0.143	0.20 ± 0.044	< 0.0001	< 0.0001	< 0.0001
*Lachnospiraceae*	12.40 ± 0.329	0.4 ± 0.063	11.80 ± 0.322	1.30 ± 0.113	< 0.0001	< 0.0001	< 0.0001
*Ruminococcaceae*	17.40 ± 0.379	0.2 ± 0.044	2.70 ± 0.162	0.40 ± 0.063	< 0.0001	< 0.0001	< 0.0001
*Erysipelotrichaceae*	0.30 ± 0.063	0.1 ± 0.031	5.20 ± 0.222	0.50 ± 0.070	< 0.0001	< 0.0001	< 0.0001
*Desulfovibrionaceae*	3.00 ± 0.170	0.2 ± 0.044	6.80 ± 0.251	0.30 ± 0.054	< 0.0001	< 0.0001	< 0.0001
*Enterobacteriaceae*	0.70 ± 0.083	47.3 ± 0.499	1.00 ± 0.099	56.8 ± 0.495	< 0.0001	NS	< 0.0001
*Bacteroidaceae*	0.20 ± 0.044	26.7 ± 0.442	0.20 ± 0.044	3.60 ± 0.186	< 0.0001	NS	< 0.0001
*S24-7*	4.10 ± 0.198	4.9 ± 0.215	1.00 ± 0.099	0.80 ± 0.089	< 0.0001	< 0.0001	NS
*Coriobacteriaceae*	1.40 ± 0.117	0.2 ± 0.044	5.10 ± 0.219	0.50 ± 0.070	< 0.0001	< 0.0001	< 0.0001
*Verrucomicrobiaceae*	0.10 ± 0.031	0.10 ± 0.031	6.40 ± 0.244	7.90 ± 0.269	NS	< 0.0001	< 0.0001
*F16*	0.10 ± 0.031	0.10 ± 0.031	11.60 ± 0.320	0.10 ± 0.031	NS	< 0.0001	< 0.0001

Values are expressed as the means ± SD. Data were analyzed by one-way ANOVA, followed by Bonferroni test, n = 6, *p* ≤ 0.05, NS = Not significant, CD: Standard—chow diet for 3 weeks; CD + A.: Standard—chow diet for 3 weeks and underwent Amoxicillin treatment in 3rd week; HSD: High-salt diet for 3 weeks; HSD + A.: High-salt diet for 3 weeks and underwent Amoxicillin treatment in 3rd week.

**Figure 7:**
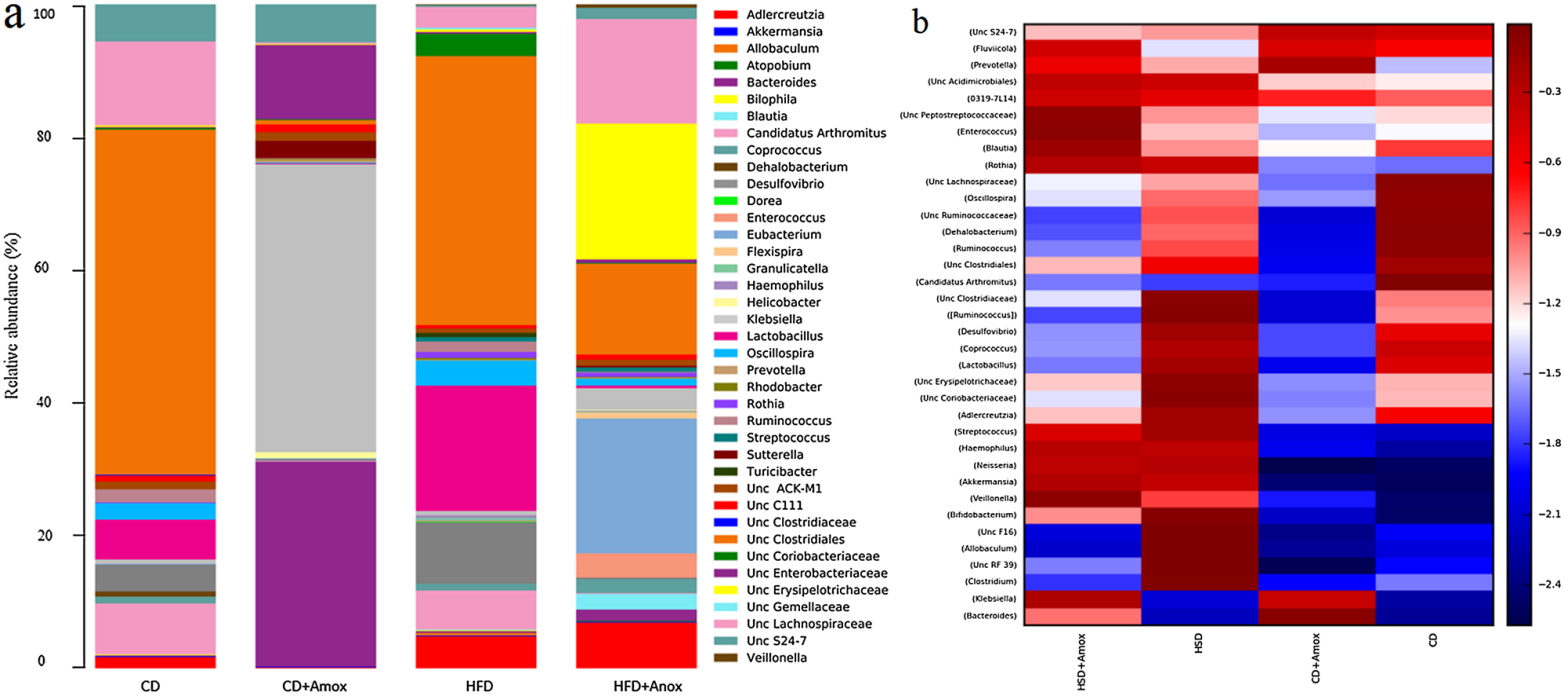
Bar chart (**a**) and heat map (**b**) showed the short-term effect of a high-salt diet (4% in chow) on the relative abundance of the most represented bacterial taxa at the genus level for each sample. Amoxicillin-treatment in HSD-fed mice found a significant decrease in *Lactobacillus, Streptococcus*, *Unc Clostridiaceae*, *Clostridium*, *[Ruminococcus]*, *Unc Erysipelotrichaceae*, *Allobaculum*, *Desulfovibrio*, *Unc Coriobacteriaceae*, *Unc F16* and a significant increase in *Bacteroides* and *Akkermansia*. The color intensity in each sample is normalized to represent its relative ratio in the four groups. The color scale of higher (dark red) and lower (dark blue) shows the relative abundances of top 36 mean abundance of bacterial communities. CD: Standard—chow diet for 3 weeks; CD + Amox: Standard—chow diet for 3 weeks and subjected to Amoxicillin treatment in 3rd week; HSD: High-salt diet for 3 weeks; HFD + Amox: High-salt diet for 3 weeks and subjected to Amoxicillin treatment in 3rd week.

We observed, at the genus level, a total of 21, 26, 44, and 47 genera (Table 7 and Fig. 7) in CD, CD + Amox, HSD, and HSD + Amox group samples, respectively. The species detected in these groups are presented in Table 8. There was a significant increase in the relative abundance of gut bacteria in HSD-fed mice such as *Lactobacillus*, *Streptococcus*, *Unc Clostridiaceae*, *Clostridium*, *[Ruminococcus]*, *Unc Erysipelotrichaceae Allobaculum*, *Neisseria*, *Desulfovibrio*, *Haemophilus, Unc Coriobacteriaceae*, *Unc F16* and *Unc RF 3*9 in HSD-fed mice (*p* < 0.0001) compared to CD-fed mice. HSD-fed mice also showed an increase in the abundance of *Ruminococcus gnavus*, *Neisseria subflava*, and a significant decrease of *Lactobacillus reuter*i (*p* < 0.0001). However, Amoxicillin treatment in HSD-fed mice caused the significant decrease in *Lactobacillus*, *Streptococcus*, *Unc Clostridiaceae*, *Clostridium*, *[Ruminococcus], Unc Erysipelotrichaceae*, *Allobaculum*, *Desulfovibrio*, *Unc Coriobacteriaceae*, and *Unc F16* (*p* < 0.0001). Amoxicillin treatment in HSD-fed mice did not cause any changes in the abundance of *Neisseria*, and *Haemophilus.* Amoxicillin-treated HSD-fed mice also showed a significant decrease in *Ruminococcus gnavus* compared to the HSD-fed mice at the species level. Even Amoxicillin-treatment to CD-fed mice also showed a significant decrease in *Lactobacillus*, *[Ruminococcus]* (*p* < 0.0001), *Unc Erysipelotrichaceae*, *Desulfovibrio* and *Unc Coriobacteriaceae* (*p* < 0.0001), (*p* < 0.0001). Amoxicillin treatment in CD-fed mice also caused a significant decrease in *Ruminococcus gnavus* (*p* < 0.0001) compared to CD-fed mice.

**Table 7:** Short term effect of high-salt (4% in chow) diet followed by Amoxicillin treatment on the mean relative abundance of major bacterial groups at genus level in mice.

Genus	Relative abundance (%)				Significance (Adjusted *p*-value)		
	CD	CD + A	HSD	HSD. + A	CD versus CD + A	CD versus HSD	HSD versus HSD + A
*Enterococcus*	0.00 ± 0.000	0.00 ± 0.000	0.00 ± 0.000	0.30 ± 0.054	NS	NS	< 0.0001
*Lactobacillus*	4.50 ± 0.207	0.10 ± 0.031	8.50 ± 0.278	0.30 ± 0.054	< 0.0001	< 0.0001	< 0.0001
*Streptococcus*	0.00 ± 0.000	0.00 ± 0.000	0.50 ± 0.070	0.20 ± 0.044	NS	< 0.0001	< 0.0001
*Unc Clostridiales*	39.00 ± 0.487	0.6 ± 0.077	15.50 ± 0.361	4.60 ± 0.209	< 0.0001	< 0.0001	< 0.0001
*Unc Clostridiaceae*	0.10 ± 0.031	0.00 ± 0.000	0.50 ± 0.070	0.00 ± 0.000	NS	< 0.0001	< 0.0001
*Candidatus Arthromitus*	5.70 ± 0.231	0.1 ± 0.031	0.10 ± 0.031	0.10 ± 0.031	< 0.0001	< 0.0001	NS
*Clostridium*	0.00 ± 0.000	0.00 ± 0.000	1.40 ± 0.117	0.00 ± 0.000	NS	< 0.0001	< 0.0001
*Dehalobacterium*	0.60 ± 0.077	0.00 ± 0.000	0.10 ± 0.031	0.00 ± 0.000	< 0.0001	< 0.0001	< 0.0001
*Unc Lachnospiraceae*	9.50 ± 0.293	0.30 ± 0.054	1.00 ± 0.099	0.50 ± 0.070	< 0.0001	< 0.0001	0.0008
*Blautia*	0.10 ± 0.031	0.00 ± 0.000	0.00 ± 0.000	0.30 ± 0.054	NS	0.0007	< 0.0001
*Coprococcus*	0.80 ± 0.089	0.00 ± 0.000	1.00 ± 0.099	0.10 ± 0.031	< 0.0001	0.0016	< 0.0001
*[Ruminococcus]*	1.10 ± 0.104	0.10 ± 0.031	9.60 ± 0.294	0.20 ± 0.044	< 0.0001	< 0.0001	< 0.0001
*Unc Peptostreptococcaceae*	0.00 ± 0.000	0.00 ± 0.000	0.10 ± 0.031	0.60 ± 0.077	NS	0.0077	< 0.0001
*Unc Ruminococcaceae*	12.10 ± 0.326	0.10 ± 0.031	2.10 ± 0.143	0.30 ± 0.054	< 0.0001	< 0.0001	< 0.0001
*Oscillospira*	1.80 ± 0.132	0.10 ± 0.031	0.30 ± 0.054	0.10 ± 0.031	< 0.0001	< 0.0001	0.0028
*Ruminococcus*	1.90 ± 0.136	0.00 ± 0.000	0.30 ± 0.054	0.10 ± 0.031	< 0.0001	< 0.0001	0.0034
*Veillonella*	0.00 ± 0.000	0.00 ± 0.000	0.10 ± 0.031	0.30 ± 0.054	NS	0.0007	< 0.0001
*Unc Erysipelotrichaceae*	0.20 ± 0.044	0.1 ± 0.031	1.90 ± 0.136	0.20 ± 0.044	0.0019	< 0.0001	< 0.0001
*Allobaculum*	0.00 ± 0.000	0.00 ± 0.000	3.10 ± 0.173	0.00 ± 0.000	NS	< 0.0001	< 0.0001
*Neisseria*	0.00 ± 0.000	0.00 ± 0.000	0.40 ± 0.063	0.40 ± 0.063	NS	< 0.0001	NS
*Desulfovibrio*	3.00 ± 0.170	0.20 ± 0.044	6.80 ± 0.251	0.30 ± 0.054	< 0.0001	< 0.0001	< 0.0001
*Klebsiella*	0.50 ± 0.070	37.5 ± 0.484	0.80 ± 0.089	50.30 ± 0.497	< 0.0001	NS	< 0.0001
*Haemophilus*	0.00 ± 0.000	0.00 ± 0.000	0.40 ± 0.063	0.40 ± .0.063	NS	< 0.0001	NS
*Bacteroides*	0.20 ± 0.044	26.7 ± 0.442	0.20 ± 0.044	3.60 ± 0.186	< 0.0001	NS	< 0.0001
*Prevotella*	0.00 ± 0.000	0.50 ± 0.070	0.10 ± 0.031	0.20 ± 0.044	< 0.0001	0.0002	0.0002
*Unc S24-7*	4.10 ± 0.198	4.90 ± 0.215	1.00 ± 0.999	0.80 ± 0.089	< 0.0001	< 0.0001	NS
*Fluviicola*	0.10 ± 0.031	0.20 ± 0.044	0.00 ± 0.000	0.20 ± 0.044	0.0019	0.0002	< 0.0001
*Unc Acidimicrobiales*	0.10 ± 0.031	0.10 ± 0.031	0.60 ± 0.077	0.70 ± 0.083	NS	< 0.0001	NS
*Rothia*	0.00 ± 0.000	0.00 ± 0.000	0.40 ± 0.063	0.50 ± 0.070	NS	< 0.0001	0.0184
*Bifidobacterium*	0.00 ± 0.000	0.00 ± 0.000	0.20 ± 0.044	0.00 ± 0.000	NS	< 0.0001	< 0.0001
*Unc Coriobacteriaceae*	0.10 ± 0.031	0.00 ± 0.000	1.60 ± 0.125	0.10 ± 0.031	< 0.0001	< 0.0001	< 0.0001
*Adlercreutzia*	1.30 ± 0.113	0.1 ± 0.031	3.40 ± 0.181	0.40 ± 0.063	< 0.0001	< 0.0001	< 0.0001
*0319-7L14*	0.00 ± 0.000	0.00 ± 0.000	0.10 ± 0.031	0.10 ± 0.031	NS	< 0.0001	NS
*Akkermansia*	0.00 ± 0.000	0.10 ± 0.031	6.40 ± 0.244	7.80 ± 0.268	< 0.0001	< 0.0001	< 0.0001
*Unc F16*	0.10 ± 0.031	0.10 ± 0.031	11.60 ± 0.320	0.10 ± 0.031	NS	< 0.0001	< 0.0001
*Unc RF 39*	0.00 ± 0.000	0.00 ± 0.000	0.50 ± 0.070	0.00 ± 0.000	NS	< 0.0001	< 0.0001

Values are expressed as the means ± SD. Data were analyzed by ANOVA, followed by Bonferroni test, n = 6, *p* ≤ 0.05, NS = Not significant, CD: Standard—chow diet for 3 weeks; CD + A.: Standard—chow diet for 3 weeks and underwent Amoxicillin treatment in 3rd week; HSD: High-salt diet for 3 weeks; HSD + A.: High-salt diet for 3 weeks and underwent Amoxicillin treatment in 3rd week.

**Table 8:** Short term effect of high-salt (4% in chow) diet followed by Amoxicillin treatment on the mean relative abundance of major bacterial groups at the species level in mice.

Species	Relative abundance (%)				Significance (Adjusted *p*-value)		
	CD	CD + A	HSD	HSD + A	CD versus CD + A	CD versus HSD	HSD versus HSD + A
*Paenibacillus*	0.0 ± 0.000	0.0 ± 0.000	0.2 ± 0.044	0.1 ± 0.039	NS	< 0.0001	0.0004
*Granulicatella*	0.0 ± 0.000	0.0 ± 0.000	0.1 ± 0.039	0.1 ± 0.039	NS	0.0002	NS
*Enterococcus casseliflavus*	0.0 ± 0.000	0.0 ± 0.000	0.0 ± 0.000	0.3 ± 0.054	NS	NS	< 0.0001
*Lactobacillus reuteri*	0.2 ± 0.044	0.0 ± 0.000	0.0 ± 0.000	0.0 ± 0.000	< 0.0001	< 0.0001	NS
*Blautia producta*	0.1 ± 0.039	0.0 ± 0.000	0.0 ± 0.000	0.2 ± 0.044	< 0.0001	0.0004	< 0.0001
*[Ruminococcus] gnavus*	1.1 ± 0.104	0.1 ± 0.039	9.6 ± 0.294	0.2 ± 0.044	< 0.0001	< 0.0001	< 0.0001
*Veillonella dispar*	0.0 ± 0.000	0.0 ± 0.000	0.1 ± 0.039	0.3 ± 0.054	NS	0.0013	< 0.0001
*Eubacterium dolichum*	0.1 ± 0.039	0.1 ± 0.039	0.2 ± 0.044	0.3 ± 0.054	NS	0.0057	0.0057
*Brevundimonas diminuta*	0.0 ± 0.000	0.0 ± 0.000	0.1 ± 0.039	0.1 ± 0.039	NS	0.0002	NS
*Neisseria subflava*	0.0 ± 0.000	0.0 ± 0.000	0.3 ± 0.054	0.3 ± 0.054	NS	< 0.0001	NS
*Haemophilus parainfluenzae*	0.0 ± 0.000	0.0 ± 0.000	0.3 ± 0.054	0.4 ± 0.063	NS	< 0.0001	0.0068
*Bacteroides acidifaciens*	0.1 ± 0.039	18.1 ± 0.385	0.1 ± 0.039	1.0 ± 0.099	< 0.0001	NS	< 0.0001
*Bacteroides uniformis*	0.0 ± 0.000	0.0 ± 0.000	0.0 ± 0.000	2.2 ± 0.146	NS	NS	< 0.0001
*Prevotella melaninogenica*	0.0 ± 0.000	0.0 ± 0.000	0.0 ± 0.000	0.2 ± 0.044	NS	NS	< 0.0001
*Candidatus Microthrix parvicella*	0.0 ± 0.000	0.0 ± 0.000	0.0 ± 0.000	0.1 ± 0.039	NS	NS	< 0.0001
*Candidatus Aquiluna rubra*	0.0 ± 0.000	0.0 ± 0.000	0.1 ± 0.039	0.0 ± 0.000	NS	< 0.0001	< 0.0001
*Rothia mucilaginosa*	0.0 ± 0.000	0.0 ± 0.000	0.4 ± 0.063	0.5 ± 0.070	NS	< 0.0001	0.0184
*Bifidobacterium pseudolongum*	0.0 ± 0.000	0.0 ± 0.000	0.2 ± 0.044	0.0 ± 0.000	NS	< 0.0001	< 0.0001
*Akkermansia muciniphila*	0.0 ± 0.000	0.0 ± 0.000	6.4 ± 0.244	7.8 ± 0.268	NS	< 0.0001	< 0.0001

Values are expressed as the means ± SD. Data were analyzed by one-way ANOVA, followed by Bonferroni test, n = 6, *p* ≤ 0.05, NS = Not significant, CD: Standard—chow diet for 3 weeks; CD + A: Standard—chow diet for 3 weeks and subjected to Amoxicillin treatment in 3rd week; HSD: High-salt diet for 3 weeks; HSD + A: High-salt diet for 3 weeks and subjected to Amoxicillin treatment in 3rd week.

The HSD-fed mice did not show any alteration in the abundance of *Bacteroides* but increased the abundance of *Akkermansia (p* < 0.0001) compared to the CD- fed mice. However, Amoxicillin-treated HSD-fed mice caused a significant increase in *Bacteroides* (*p* < 0.0001) and *Akkermansia* (*p* < 0.0001). In this study, Amoxicillin treatment in HSD-fed mice also resulted in a significant increase in *Bacteroides acidifaciens*, *Bacteroides uniformis* (*p* < 0.0001), and *Akkermansia muciniphila* (*p* < 0.0001) linked to an increase in insulin sensitivity and gut epithelial integrity. Amoxicillin-treated CD-fed mice also showed a significant increase in *Bacteroides acidifaciens* and *Akkermansia muciniphila* (*p* < 0.0001) compared to the CD-fed mice.

There was a significant increase in the relative abundance of gut opportunistic pathogens such as *Veillonella* (*p* = 0.0007), *Prevotella* (*p* = 0.0002), *Rothia* (*p* < 0.0001) in HSD-fed mice compared to CD-fed mice. HSD-fed mice also showed a significant increase in *Veillonella dispar* (*p* = 0.0013), *Rothia mucilaginosa* (*p* < 0.0001), a) compared to the CD-fed mice. However, Amoxicillin treatment in HSD-fed mice caused a significant increase in *Veillonella* (*p* < 0.0001), *Klebsiella* (*p* < 0.0001), *Prevotella* (*p* = 0.0002), *Rothia* (*p* = 0.0184), and *Enterococcus* (*p* < 0.0001)). Amoxicillin treated HSD-fed mice also showed a significant increase in *Veillonella dispar* (*p* < 0.0001), *Rothia mucilaginosa* (*p* < 0.0184), and *Enterococcus casseliflavus* (*p* < 0.0001). Similarly, amoxicillin treatment in CD-fed mice also resulted in a significant increase in *Veillonella*, *Klebsiella* (*p* < 0.0001), *Prevotella* (*p* = 0.0002), and *Rothia* (*p* < 0.0001).

Taken together, our result indicates that HSD for a short period of 3 weeks in mice could significantly change the gut microbiota profile (phylum, family and genus level) known to develop the pathophysiological features of metabolic syndrome-related inflammation. Importantly, our investigation observed that Amoxicillin treatment in HSD-fed mice causes reshaping of specific gut bacteria associated with improvement in the pathophysiological attributes of metabolic disease related inflammation, during the consumption of HSD in mice.

**Figure 8:**
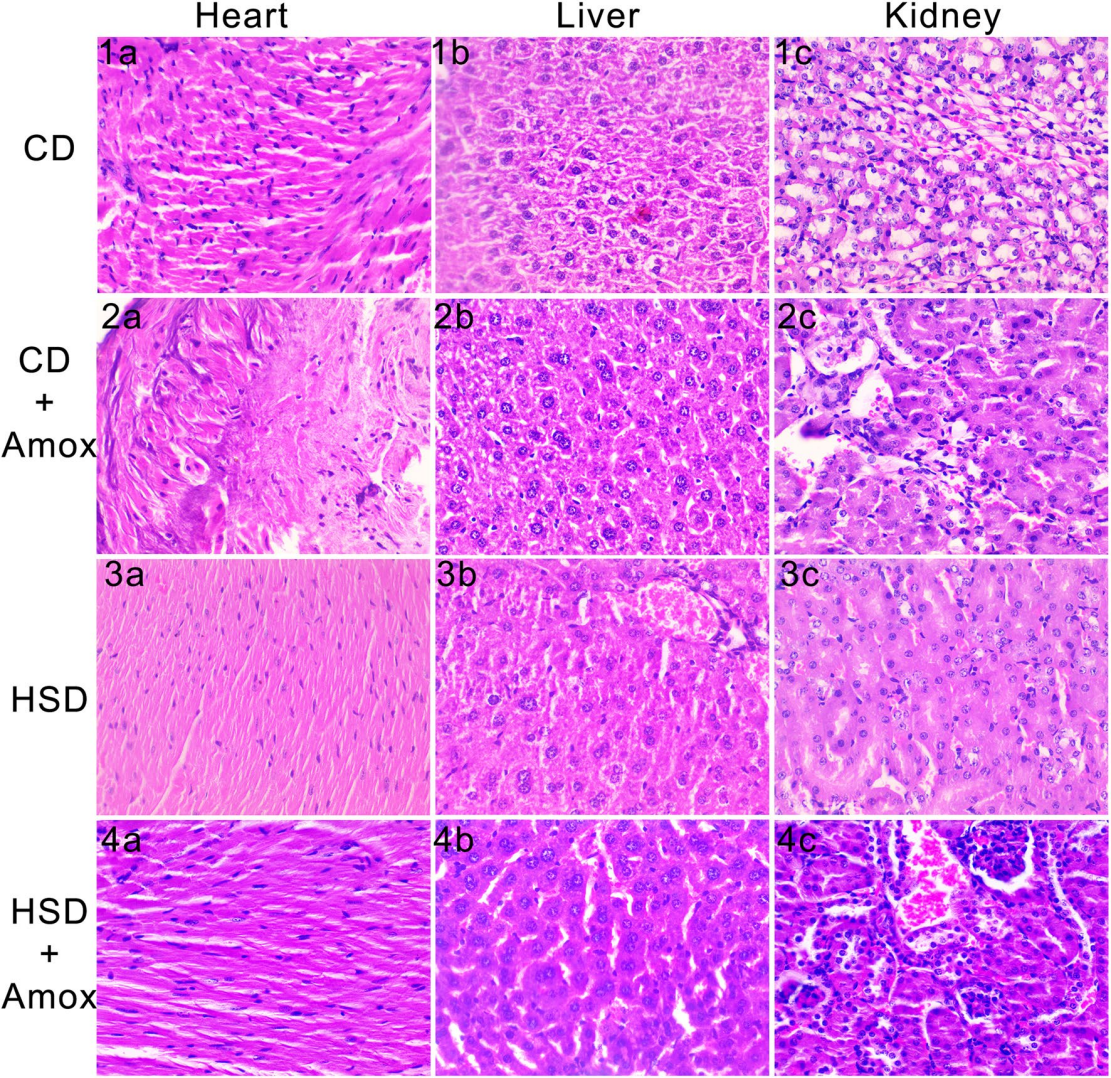
Short-term effect of high-salt diet (4%) followed by Amoxicillin treatment on the heart, liver, and kidney of mice. The figure represents histopathogical examination of heart, liver and kidney sections by hematoxylin and eosin staining. The photomicrograph of the heart tissue of HSD-fed mice showed mild cardiomyocyte hypertrophy and associated degenerative changes (3a). Liver histopathology also indicated the changes in HSD-fed mice, including oddly shaped cells and decreased cell proliferation (3b). No significant changes were observed in the kidney issue of HSD-fed mice (3c). However, these histopathological changes were insufficient to conclude as the significant observations. No significant changes were observed in the heart, liver and kidney of Amoxicillin treated HSD-fed mice (4a, 4b and 4c).

We found that no significant histopathological damage occurs in CD-fed mice, HSD-fed mice and mice receiving amoxicillin treatment with these diets (Fig. 8). The photomicrograph of the heart tissue of HSD-fed mice showed mild cardiomyocyte hypertrophy and associated tissue degenerative changes (3a). Liver histopathology also indicated the changes in HSD-fed mice, including oddly shaped cells and decreased cell proliferation (3b). No significant changes were observed in the kidney tissue of HSD-fed mice (3c). However, these histopathological changes were insufficient to conclude that they were significant. No significant changes in the heart, liver, and kidney were observed in Amoxicillin treated HSD-fed mice (Fig. 4a, 4b, 4c). Our study indicates that HSD for a short period could not cause significant histopathological changes in the heart, liver and kidney of mice (Fig. 8).

## Discussion

Excessive dietary salt intake is associated with metabolic abnormalities such as hypertension, cardiovascular complications, renal injury, and gut inflammation^20,21^. A metabolic syndrome is characterized by abnormal cholesterol levels, high fasting blood sugar level, high triglyceride level, and chronic inflammation, caused primarily by modulation of the gut microbiota^22,23^. Further, it is speculated that diabetes patients who take a certain class of antibiotics are more likely to have severe blood sugar fluctuations than those who take other types of the drugs. Nevertheless, determining how antibiotics treatment in the context of HSD diet influences gut microbiota, haemato-biochemical parameters, as well as histopathological changes in vital organs, is a topic of high interest to researchers. Earlier studies focused on the detrimental effect of consuming a high amount of salt for a long period on human health^24–29^. However, less has been known about the short-term impact of an HSD on gut microbiota and associated pathophysiology. In our study, we fed mice a short-term HSD diet for 3 weeks, followed by an Amoxicillin intervention in the 3rd week.

Previous investigations have shown that HSD for the period of 4 weeks or 8 weeks did not lead to any change in the faecal bacterial richness and diversity but displayed even variability or similarity in bacterial composition within groups^7,30^. Our findings are not consistent with previous research and showed that HSD treatment in mice causes an increase in taxonomic richness and diversity^7,30^, particularly of specific gut bacteria such as *Streptococcus Clostridium, Unc Peptostreptococcaceae, Veillonella, Allobaculum, Neisseria, Prevotella, Haemophilus, Rothia, Bifidobacteria* and *Unc RF 39*, which were exclusively detected in HSD-fed mice. However, Amoxicillin treatment resulted in a reverse effect in HSD-fed mice by depletion of *Clostridium, Unc Clostridium Dehalobacterium Allobaculum, Bifidobacteria,* and *Unc RF 39.* In addition, our study also identified the different compositions of gut microbiota in each group**.**

Here, we consistently found a significant increase in the *F/B* ratio in HSD-fed mice associated with the pathophysiology of metabolic syndrome^7,31–34^. Amoxicillin treatment in HSD-fed mice substantially reduced the *F/B* ratio with a significant increase in the abundance of *Proteobacteria.* To our knowledge, this is the first study to demonstrate that HSD intake causes a substantial rise in *Desulfovibrionaceae, Erysipelotrichaceae*, and *Enterobacteriaceae,* which induce the pathophysiology of obesity and metabolic syndrome-related inflammation^35,36^. *Enterobacteriaceae* usually cause acute infective process^37,38^. Furthermore, we were also observed a significant increase in *Coriobacteriaceae* in HSD-fed mice. Previous studies reported that the occurrence of *Coriobacteriaceae* has a positive correlation with obesity and type-2 diabetes^39–41^ Furthermore, HSD-fed mice also observed the significant increase of *F16* known to be involved in the manifestation of inflammatory bowel diseases^14^. However, the treatment of Amoxicillin-in mice fed on HSD demonstrated a decrease in the abundance of *Desulfovibrionaceae, Erysipelotrichaceae*, *Coriobacteriaceae*, *F16*, and increased in the level of *Enterobacteriaceae.* Another key finding in this study was that *Bacteroidaceae* known to be negatively related to many metabolic phenotypes such as plasma insulin levels and weight gain, were exclusively increased in the Amoxicillin-treated both HSD-fed mice and CD-fed mice^42^. Unexpectedly, our study found significant increase in *Verrucomicrobiaceae* reported for a negative association with endotoxemia, adipose tissue inflammation, and insulin resistance in HSD-fed mice^34^. Amoxicillin treatment further significantly increased the abundance of *Verrucomicrobiaceae*.

Our genus-level results confirmed the abundance of gut microbiota, including *Lactobacillus, Streptococcus*, *Ruminococcus gnavus, Allobaculum*, *Desulfovibrio, Neisseria. subflava, Clostridium* and *Haemophilus* in HSD-fed mice. Most importantly, our study showed a surprise increase in the abundance of *Lactobacillus* that is not in line with the previous studies, which reported their suppression in HSD-fed mice^7,30,44^. Although the *Lactobacillus* genus is potentially probiotic bacteria, however its effect is species and even strain specific. *Lactobacillus* abundance is known to have a positive correlation with fasting blood glucose and HbA1c^45^. However, in our study, the representative species, *Lactobacillus reuteri*, which is well known to play an active role in the maintenance of the gut epithelial barrier by secreting antibiotic substances such as *reuteri, and* is also reported to have a beneficial impact on the pathophysiology of T2D, was observed for a decrease in their proportion in HSD-fed mice^46,47^. A previous study has reported a positive correlation between *Streptococcus* and metabolic disease development^48^. There is an agreement with the recent publication article by Wang, C. et al., which showed the increase in the abundance of *Ruminococcus in* HSD-fed mice^7^. Additionally, this study observed the increase of *Ruminococcus gnavus* linked to production of a polysaccharide that stimulates the release of inflammatory cytokines and induces inflammatory bowel disease^49^. Congruently, our investigation also found a higher level of *Allobaculum* in HSD-fed mice^44^. An earlier study has shown the positive association between the *Allobaculum* and the expression of *angiogenin-like protein 4* (ANGPTL4), which has a crucial role in lipid deposition by repressing the *lipoprotein lipase* (LPL) enzyme and developing the pathophysiology of the obesity-related metabolic disorder^50^. In this investigation, we also observed a higher proportion of *Desulfovibrio* known to provoke intestinal barrier dysfunction and insulin resistance^51,52^. The previous study has shown a positive correlation between *Neisseria* and plasma cholesterol^53^. Further, the increase in the abundance of *Neisseria subflava* is considered an indicator of a change in microbial community composition to become acid-secreting^54^. Most strains of *Clostridium* and *Haemophilus* are potentially pathogenic to humans^55^. In our study Amoxicillin treatment to HSD-fed mice caused a decrease in the abundance of these specific gut bacteria.

Furthermore, a previous study has shown that *Bacteroides* are capable of preventing salt induced effects due to having salt tolerant genes^56^. Similarly, this study did not show any change in the abundance of *Bacteroides* in HSD-fed mice*.* Interestingly, we found an increase in the abundance of *Bacteroides* in Amoxicillin treated HSD-fed mice. Moreover, this study observed an exclusive increase in the proportion of *Bacteroides acidifaciens* and *Bacteroides uniformis* in Amoxicillin treated HSD-fed mice*.* A previous study has shown that these gut bacteria species improve insulin sensitivity and prevent obesity by activating Glucagon*-like peptide-1 (GLP-1)*^23^. Most unexpectedly, our study showed an increase in the abundance of *Akkermansia muciniphila* known for its decrease in glucose intolerance^57,58^. Along with beneficial impact of Amoxicillin on HSD-fed mice, we also observed the abundance of some opportunistic gut pathogen bacteria such as *Klebsiella, Prevotella melanogeni, Veillonella dispar*, and *Enterococcus casseliflavus* in Amoxicillin treated HSD-fed mice. These are the opportunistic pathogens that cause the disruption in the gut^59–65^, Collectively, these results suggest that Amoxicillin treatment in mice fed HSD causes a change in specific gut microbiota associated with improving the pathophysiological attributes of metabolic disorder related inflammation however, it also puts some at risk for opportunistic pathogens.

Earlier studies have shown that the gut microbiota is a key regulator of energy homeostasis^66–68^. and their composition is related to insulin sensitivity and host glycemic regulation^47,69^. The previous researchers lend support to the view that a long-term intake of a high-salt diet is associated with the pathophysiology of metabolic syndrome^71–73^. However, some studies have demonstrated that HSD for a short period also reduced insulin sensitivity in human and rat models^74,75^. Our study found that HSD increases the blood glucose level with abundance of specific gut microbiota such as *Lactobacillu*s, *Streptococcus, Ruminococcus gnavus*, *Allobaculum, and Desulfovibrio.* These microbiotas are known for having a positive correlation with high plasma glucose level^45,48–51^. Contrary to our findings, Pitynski-Miller, D et al., reported that high-salt diets might block high–fat–diet-induced obesity either by impairing fat absorption or by unknown mechanisms^76^. Most strikingly, our data showed that Amoxicillin treatment in HSD-fed mice significantly reduced the glucose level, suggesting its hypoglycemic effect. In fact, previous studies have shown that antibiotics deplete or even reshape specific gut microbial communities involved in glucose metabolism^77,78^. Our study elucidates a significant suppression of *Streptococcus, Lactobacillu*s, *Ruminococcus gnavus*, *Allobaculum, Desulfovibrio*, and enrichment of Bacteroides *acidifaciens*, *Bacteroides uniformis*, and *Akkermansia muciniphila* in Amoxicillin-treated HSD-fed mice. Incongruent to Seck et al., our result revealed the exceptionally enrichment of *Akkermansia muciniphila* reported for improving glucose tolerance in HSD-fed mice^79,80^. These findings suggest that Amoxicillin-treatment in HSD-fed mice results in the restoration of glucose levels near to true values by substantially changing the composition of these gut bacteria. Although, the conclusion of the study opens the door for future research to find out which specific gut microbiota’s metabolites involve in the restoration of blood glucose levels towards the normal range by improving the insulin signaling in the mice.

High salt consumption increases cardiovascular diseases (CVDs) risk by impairing endothelial function, most notably by reducing nitric oxide (NO) availability^81,82^. The increase in total cholesterol levels in short term is associated with significant decreases in endothelium-dependent vasodilation^83,84^. Metabolic factors, such as high glucose and triglyceride levels, are considered as major risk factors in contributing to endothelial dysfunction by decreasing nitric oxide absorption^85,86^. In our study HSD-fed mice showed increased levels of serum triglycerides and significant increases in glucose levels. The increase in these metabolites is likely to cause vascular endothelial dysfunction and may account for the short-term increase in total cholesterol in mice fed HSD. Importantly, our data consistently showed that even short-term HSD exposure to mice could result in increase the cholesterol level significantly along, with significant change in microbiota composition^87^. There are further evidences that the gut microbiota has the capacity to alter the blood lipid composition, particularly cholesterol, through gut bacteria related metabolites^68,88–90^. In fact, it is well known that the intestinal gut bacteria determine the circulating cholesterol level. Previous studies have shown that *Erysipelotrichaceae* and *Coriobacteriaceae* have a positive correlation with high plasma cholesterol in human and animal models^89,91–94^. The gut bacteria are directly involved in the pathogenesis of cardiovascular disease (CVD) through the alteration of circulating cholesterol levels. We also found the higher abundance of specific gut microbiota such as *Lactobacillus, Enterobacteriaceae*, *Streptococcus* and increased *F/B ratio* reported with cardiovascular diseases (CVD)^95^. Moreover, one study has shown a positive correlation between Neisseria and plasma cholesterol^53^. Interestingly, we were also able to detect similar modulation in the composition of gut microbiota and we believe that this may be the likely cause of increasing the plasma cholesterol in HSD-fed mice. However, the investigation to find out which gut bacteria metabolites are involved in the increase in the level of cholesterol is the topic of future research interest. We further believe that Amoxicillin treatment in HSD-fed mice could improve endothelial function by lowering serum triglyceride and glucose levels, which could lead to a reduction in cholesterol levels. Importantly, the treatment with Amoxicillin resulted in almost the suppression of these specific gut bacteria except for the enrichment of *Enterobacteriaceae* in HSD-fed mice. Amoxicillin treated HSD-fed mice however showed a slight but not significant reduction in cholesterol levels.

Our research consistently revealed the increase in creatinine levels in HSD-fed mice^96^. Many studies have indicated that high salt intake induces the pathophysiology of progressive impairment in renal function either through BP-dependent or independent mechanisms^97–99^. A high sodium intake impairs vascular function primarily by decreasing nitric oxide bioavailability. However, prostaglandins, adenosine, pH, PO2, and myogenic factors have been reported as major regulators of this response^100,101^. In addition, the metabolic factors, such as glucose and triglycerides at high level can also contribute to endothelial dysfunction by increasing oxidative stress and decreasing nitric oxide bioavailability^96^. Furthermore, our study found an increase in serum triglycerides and a significant increase in glucose levels in HSD-fed mice, which may result in endothelial remodelling causing adverse effects on renal hemodynamics by reducing glomerulus filtration efficiency, which in turn could increase blood creatinine levels. In our study we found that Amoxicillin treatment in HSD-fed mice caused a significant decrease in serum triglycerides and glucose toward the control values that could improve vascular endothelial function and result in an increase in the glomerular filtration rate and decrease in serum creatinine levels in mice. Here, we provide an insightful clinical finding that Amoxicillin treatment in HSD-fed mice significantly reduced plasma creatinine levels to a control range.

Consistently, our data clearly showed a decrease in plasma urea in HSD-fed mice^102^. According to a previous study, ammonia in the gut lumen is not the result of host urease activity, but rather of proteolytic and microbial urease activity^103^. Indeed, HSD caused decreased digestion of protein as well as reduced transit time in the digestive tract by modulation of specific gut bacteria^7^. Further, the increase of protein in urine excretion was also associated with the intake of high salt intake^104^. We believe the poor absorption of protein in the intestine and loss through urine could be the probable reason for a low level of blood urea in HSD-fed mice. Notably, Amoxicillin treatment in HSD-fed mice restored blood urea concentration to the control range. Similarly, Amoxicillin treatment in CD-fed mice also decreased plasma urea levels. A previous study observed that oral antibiotic administration affected urea kinetics by increasing urine excretion and increasing blood concentration of urea N by affecting N metabolism^105^. In the intestine, *Bacteroides* spp. secrete proteases at the brush border of absorptive cells, leading to higher levels of ammonia^105^. Our study also found an increase in the abundance of *Bacteroides* spp. for both HSD-fed mice and CD-fed mice that could be the probable cause of the increase in serum urea. The present study also consistently observed the effect of HSD on blood cells especially the increase in the concentration of thrombocytes in mice^106^. However, Amoxicillin treatment in CD-fed mice did not alter haematological parameters substantially. One study found that HSD significantly increased *IL-6* production and induced the platelet production^107,108^. Interestingly, Amoxicillin treatment brought the elevated levels of the thrombocytes in HSD-fed mice closer to the true levels. Based on these observations, we could suggest that Amoxicillin treatment restores thrombocytes value by decreasing the level of specific cytokines such as *IL-6*. Accordingly Amoxicillin appears to be able to reduce the risk of clotting, stroke or heart attack to the individual on HSD. However, more advanced studies are needed to pinpoint the underlying mechanism by which Amoxicillin treatment in HSD-fed mice reduced the level of the thrombocytes to control levels.

Several studies have found that long term intake of HSD leads to obesity, and the pathophysiology of metabolic syndrome^71–73^. According to a study conducted by Dobrian, et al. on the laboratory rats by providing an HSD for a long duration caused obesity and an increasing adipocyte size^109^. However, we conducted a short-term study by providing HSD for a period of 3 weeks and did not find any significant gain in the weight of mice. Our results are consistent with the established observations in the field which demonstrate that a high-salt diet has been reported to cause hyperphagia in mice and humans, but body weights remained similar acutely (over 4 wks.) under ad libitum dietary conditions due to a hypercatabolic state^110,111^.

Many studies have provided extensive evidence that HSD consumption promotes the development of hypertrophy as well as fibrosis in the heart and kidney by overexpressing cytokines, namely transforming growth factor-1 (TGF-1)^112–114^. Also another study found that HSD cause the excess production of reactive oxygen species (ROS) which causes liver fibrosis^115^. This study observed only mild cardiomyocyte hypertrophy and associated tissue degenerative changes, oddly shaped cells, and a decrease in cell proliferation in the heart of the HSD-fed mice. However, these small pathological findings did not lead to any significant conclusions. Further, mice fed HSD did not develop any significant pathological changes in the kidney. Our study suggests that short term intake of HSD did not cause any significant pathological progression in the heart, liver and kidney of mice. Any significant pathological changes were not observed in the heart, liver and kidney of Amoxicillin treated HSD-fed mice. We believe that Amoxicillin treatment showed beneficial impact by reducing the minimal pathological changes observed in the heart of HSD-fed mice by inhibiting the upregulation of transforming growth factor as also reported by Melhus et al.^116^.

In summary, the present study showed that Amoxicillin treatment has a positive impact on a high salt diet as shown by haematology and serum biochemistry findings. There was a significant reshaping of the gut microbiota ascribed to their beneficial impact by improving the biochemical and physiological outcomes. However, this study has some limitations like the use of mouse models. Therefore, the data obtained from this study may not be directly extrapolated to humans and need to be validated. Further, we decreased the faecal samples per group and examined their relative proportions in the total bacterial population for cost-effectiveness. Taken together, our study findings provide proof of concept that Amoxicillin has a positive impact on the pathophysiological attributes of metabolic disease related inflammation collectively by improving biochemical health and gut health. We believe that long term exposure to HSD is necessary to induce the complete pathophysiology of metabolic disorders in the host. Therefore, further studies would be required to evaluate the long-term effects of antibiotic administration in mouse models of metabolic syndrome, especially in the context of diabetics.

## Data Availability

All data generated or analysed during this study are included in this manuscript. Metadata is available at: https://dataview.ncbi.nlm.nih.gov/object/PRJNA821450?reviewer=spn10eelulrond90jtemfqqpkg in read-only format.

## Abbreviations

ANGPTL4: Angiopoietin-related protein
CD: Standard chow diet
CD + Amox: Standard chow diet + Amoxicillin
CVDs: Cardiovascular diseases
FDA: Food and Drug Administration
GPR4: G Protein-Coupled Receptor 4
HSD: High-salt diet
HSD + Amox: High-salt diet + Amoxicillin
HbA1c: Haemoglobin A1C
IL-6: Interleukin 6
LPL: Lipoprotein lipase
Olfr78: Olfactory receptor-78
OTUs: Operating taxonomic units
PCoA: Principal coordinates analysis
ROS: Reactive Oxygen Species
SCFAs: Short-chain fatty acids
TMAO: TrimethylAmine -n-Oxide
T2D: Type 2 diabetes
TGF-β1: Transforming growth factor-β1
WBC: White blood cells
NO: Nitric oxide
